# Isorhynchophylline alleviates cartilage degeneration in osteoarthritis by activating autophagy of chondrocytes

**DOI:** 10.1186/s13018-023-03645-4

**Published:** 2023-03-02

**Authors:** Jieyun Jiang, Jin Li, Chenwei Xiong, Xindie Zhou, Ting Liu

**Affiliations:** 1 Medical Development Department, The Second Affiliated Hospital of Jiaxing University, Jiaxing, 314000 China; 2 Department of Orthopedic Surgery, The Second Affiliated Hospital of Jiaxing University, Jiaxing, 314000 China; 3 Department of Orthopedics, The Affiliated Changzhou Second People’s Hospital of Nanjing Medical University, Changzhou, 213000 China; 4 Changzhou Medical Center, Nanjing Medical University, Changzhou, 213000 China; 5 Department of Orthopedics, Gonghe County Hospital of Traditional Chinese Medicine, Hainan Tibetan Autonomous Prefecture, 811800 Qinghai Province China

**Keywords:** Isorhynchophylline, Chondrocytes, Osteoarthritis, Autophagy, PI3K/AKT/mTOR signaling

## Abstract

**Context:**

Osteoarthritis is a common degenerative disease, the cause of it is still unknown, and the treatment mainly focuses on improving symptoms. Studies have found that Isorhynchophylline (Isorhy) has antioxidant, anti-inflammatory, antiproliferative and neuroprotective effects.

**Objective:**

This study investigates the role and mechanism of Isorhy in OA.

**Methods:**

The destabilized medial meniscus model was used to mimic OA. Fifteen male Sprague Dawley rats were partitioned into three portions: Normal group, OA group (surgery; normal saline treatment) and OA + Isorhy group (surgery; 50 μM Isorhy treatment) were performed on the first day of every week from the 5th to the 8th week after surgery. After 4 weeks of drug treatment, the rats have been processed without debridement of the knee specimens and fixed using 4% paraformaldehyde for two days. The morphological analysis was performed by H&E, Safranin O-Fast green staining and micro-CT analysis. The specimens were researched employing Micro-CT. In the part of the aggregate methods that were evaluated by qRT-PCR and western blot of the following proteins LC3II/LC3I, Beclin-1, ATG5, ATG7, MMP3 andMMP13. Akt/PI3K signaling related proteins (p-AKT, AKT, p-PI3K, PI3K, p-mTOR, mTOR) were detected by Western blot. BECLIN1 and MMP3 were detected by Immunofluorescence assay.

**Results:**

In this present research, it was proved that autophagy-related and cartilage matrix-related proteins in osteoarthritis could be regulated by Isorhynchophylline treatment. The transcriptome sequencing results suggested the regulation was closely associated with PI3K/AKT/mTOR pathway, thereby alleviating osteoarticular inflammation. In-depth study showed that Isorhy could also affect OA in rat OA models, that was indicated by H&E, Safranin O-Fast green staining, and also micro-CT analysis.

**Conclusion:**

Our findings indicated that Isorhy could be regarded as a prospective candidate for OA treatment.

## Introduction

Osteoarthritis [1] is a familiar chronic joint disease that manifests itself as degenerative changes in articular cartilage (changes similar to aging of machine parts) and secondary osteophytes [2–4]. According to statistics, OA has become one of the most common chronic diseases in the world, affecting about ten percent of men and eighteen percent of women more than the age of 60, and recent studies have shown a trend toward younger onset of OA [5, 6]. In addition, it is noteworthy that the pathogenesis of OA is still unclear, and it is urgent to investigate its pathogenesis and effective treatment methods. Existing studies have shown that a decrease in chondrocytes' ability to repair tissues, mitosis and synthesis, and a decrease in responsiveness to anabolic growth factors are the key reasons for the development of OA [7, 8]. Therefore, it is important to investigate the factors affecting chondrocyte mitotic and metabolic activities so as to reveal the molecular mechanisms of OA and seek therapeutic options.

Isorhynchophylline (Isorhy) is a hydroxyindole alkaloid with strong pharmacological activity extracted from Uncaria rhynchophylla, a traditional Chinese medicine of Rubiaceae [9–11]. At present, it has been used in clinical treatment of cardiovascular diseases and central nervous system diseases [9–11]. It has shown a wide range of pharmacological effects and a variety of biological activities, such as the protective effect on neuronal damage induced by ischemia [12, 13]; Inhibit the release of astrocyte inflammatory factors induced by lipopolysaccharide; It significantly reduces the excitability of cerebral cortex and has obvious effects of sedation, sleeping, hypotension and spasmolysis. It is one of the main components of oral liquids [12, 13]. These studies show that Isorhy has a wide range of pharmacological effects in various diseases from different angles. Few researches have investigated its effect in osteoarthritis and cellular autophagy.

Autophagy is an intracellular homeostatic mechanism that plays a key word in maintaining intracellular environmental homeostasis, promoting cell survival by participating in the clearance of damaged organelles and the recycling and reuse of intracellular macromolecules [14, 15]. The role of chondrocyte autophagy in osteoarthritis autophagy is a self-degradation process, which refers to the self-protection mechanism of cells to survive by degrading their own structure or substances. It acts as an important part in maintaining the balance of body metabolism, and can regulate the growth and differentiation of cells. The level of autophagy is protective or damaging to the body depends mainly on the nature of the stress, the type of cell, and the state. When apoptosis is ineffective, by inducing excessive autophagy, it may also be the main cell death mechanism. In this context, autophagy directly regulates cell death mechanisms to promote death [16]. In recent years, it has been found that the course of OA is along with abnormal changes in the level of cartilage autophagy. First of OA, chondrocytes respond to cartilage damage caused by changes in the surrounding microenvironment by increasing the level of autophagy, but as OA progresses, the level of chondrocyte autophagy decreases significantly, which becomes one of the important factors for accelerated chondrocyte apoptosis and cartilage degeneration.

Autophagy can be regulated through various signaling pathways, the most important of which is mTOR signaling. The mTOR signaling is a highly conserved protein kinase that is a part of the PI3K protein kinase family and takes a downstream effector protein of the PI3K/AKT signaling pathway [17]. There are three known pathways upstream of mTOR, namely growth factor signaling pathway, energy change pathway and activation pathway of mTOR by certain types of amino acids [18]. The PI3K/AKT/mTOR signaling pathway is the principal part of the growth factor signaling pathway in mTOR, which is closely related to cell transcription, translation, growth, autophagy and apoptosis.

The objective of this study is to investigate whether Isorhy can regulate autophagy, protecting chondrocytes with OA and to determine the signaling pathway related to this. Besides, the related signaling pathway would be explored. All of these would provide a theoretical reference for the clinical practice of Isorhy in OA treatment.

## Materials and methods

### Cell culture and induction

The normal human chondrocyte cell line (C28/I2) were incubated in a culture medium containing 90% high glucose DMEM (Dulbecco’s modified eagle medium, Hy Clone, USA) and 10% Fetal Bovine Serum (FBS, Gibco, USA) at 37 degrees Celsius and 5% CO_2_. A cellular model of OA was stimulated with 10 ng/mL IL-1β induced in C28/I2 chondrocytes.

### Cell viability assay

The influences of Isorhy at concentrations of 5, 10, 20, 25, 50, 100 and 200 μM on the cell viability of normal and OA chondrocytes were investigated by Cell Counting Kit 8. Chondrocytes inoculated into 96-well plates at a density of 5000 cells/well were treated without or with different concentrations of Isorhy for 48 h. Then, the concentration 10 μL CCK-8 was putted into each well. After 2 h of culture, the optical density (OD) value was measured at 490 nm.

### qRT-PCR detection

Total RNA was extracted from each group of cells using Trizol reagent. RNA was obtained through incubation in chloroform solution for 15 min at room temperature followed by centrifugation with isopropanol. cDNA was obtained by reverse transcription according to the kit manufacturer's instructions. qRT-PCR was then performed according to the instructions to analyze the expression of LC3II/LC3I, Beclin-1, ATG5, ATG7, MMP3, MMP13 expression. mRNA cycling conditions: firstly 95 °C for 30 s, secondly 40 cycles of 95 °C for 5 s, thirdly 55 °C for 30 s, fifthly 72 °C for 1 min. Data analysis of the target genes was performed using the 2^−ΔΔCt^ method.

### Western blotting

Cells of the exponential growth phase were collected. The collected chondrocytes were treated with PBS buffer (Invitrogen, USA) according to the kit manufacturing instructions to extract total protein from each group of cells. Then, protein concentrations were calculated using the BCA protein assay kit. Each protein sample was mixed with 12% SDS-PAGE protein buffer, boiled in a water bath to denature the proteins, and appropriate amounts of each group of proteins were transferred to a polyvinylidene difluoride membrane and incubated with primary antibodies (including LC3II/LC3I, Beclin-1, ATG5, ATG7, MMP3, MMP13, and p-AKT, AKT, p-PI3K, PI3K, p-mTOR, mTOR, and GAPDH), GAPDH was used as a negative control. Finally, the results obtained were analyzed using Image J and Graphpad prism software.

### Immunofluorescence assay

The chondrocytes of the different groups were cleaned thrice with PBS and fixed with cold methanol for 20 min. The cells were infiltrated with 0.1% Triton X-100.The cells were then treated at 4 °C for 24 h with first-degree antibodies against BECLIN1 or MMP3.A second-order Alexa Fluor 594 binding antibody was added and maintained at 25 °C for 1 h. The nuclei were stained with DAPI for 5 min. Cytoskeleton was stained with Phalloidin labeled with GFP. Then, the Fluorescence microscope was used to estimate the fluorescence intensity.2.6 RNA-sequencing.

The cells were treated into three groups: control group, OA group, and OA + 25 μM Isorhy, and RNA sequencing was performed to decide the effects in the mRNA expression profiles. Total RNA was isolated from chondrocytes by TRIzol method. The obtained total RNA was subjected to RNA-seq operation on BGISEQ-500. The sequencing results were further analyzed using the R language program to search for differential genes in the microarray lists. What’s more, Gene Ontology (GO) and Kyoto Encyclopedia of Genes and Genomes (KEGG) pathway enrichment analysis were accurate applications to the R program.

### Animal model

The destabilized medial meniscus (DMM) model was used to mimic OA. 15 four-week-old male Sprague Dawley rats (200–250 g) were correctly partitioned into three portions: Normal group, OA group (surgery; normal saline treatment on the Monday of every week from the 5th to the 8th week after surgery) and OA + Isorhy group (surgery; 50 μM Isorhy treatment on the first day of every week from the 5th to the 8th week after surgery). After 4 weeks of drug treatment, all animals were euthanized through CO_2_ induction. Then the rats had been processed without debridement of the knee specimens and fixed using 4% paraformaldehyde for two days. This study was conducted according to the NIH guidelines (NIH Pub No 85-23, revised 1996) and the Chinese Regulations for the Management of Laboratory Animals, and followed the Guide for the Care and Use of Laboratory Animals published by the National Institutes of Health (NIH) in 2011, which has already been permitted through the ethics committee of the Second Affiliated Hospital of Jiaxing University.

### Micro-CT analysis

The SD rats were killed, these rats’ femoral specimens were gathered and developed in 10% formalin solution. The specimens were researched employing Micro-CT with the bellowing parameters: 18 µm, 65 kV, 385 mA. Three-dimensional reconstruction of scanned femoral images using simulation software. Use the Dataview and CT analyzer software to specify an appropriate 3 mm cylindrical area as the region of interest (Roi).

### Histological analysis and immunohistochemistry.

Knee joint tissue cells were taken from each group of mice and developed in 4% paraformaldehyde lasting for 48 h. Then, they were decalcified in 10% EDTA solution for 10 days. Finally, after dehydration in ethanol and embedding in paraffin, 10 μm sample specimens were sectioned. The specimens were stained with HE using hematoxylin and eosin (H&E) and safranin-O fast green for morphological analysis. The specimens were fixed, dehydrated, paraffinized, embedded, sectioned, then dewaxed, dehydrated, stained, sealed, and finally finished staining according to the kit instructions and pathological examination standards, and the results were analyzed taking a Canon microscope imaging system (model EOS-350D, Canon, Tokyo, Japan).

### Statistical analyses

Statistically, the experimental data were handled by SPSS 20.0 (IBM Corp., Armonk, NY, USA) and GraphPad 7 (GraphPad Software, San Diego, CA, USA). These student's tests were applied for comparisons during groups. What’s more, one-way ANOVA was applied for various sets comparisons.

## Results

### Cytotoxicity of Isorhy on chondrocytes

**Fig. 1:**
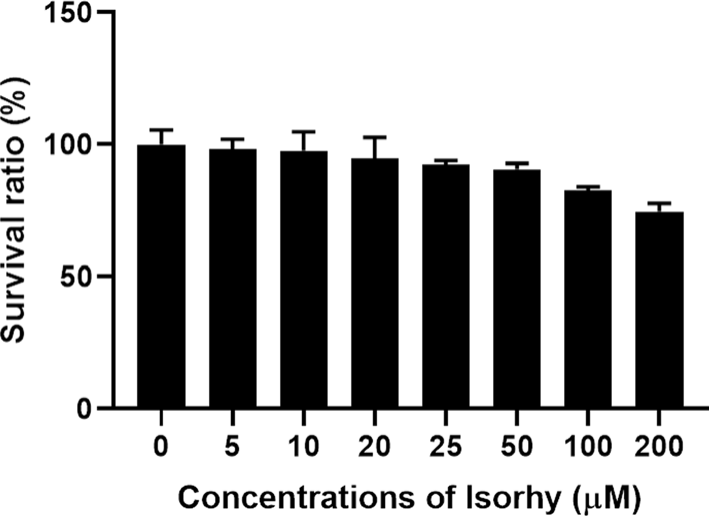
The results of cell viability and cytotoxicity of chondrocytes treated with different concentrations of Isorhy. The cartilage cells were treated with various Isorhy (0, 5, 10, 20, 25, 50, 100 and 200 μM) of UA for 48 h

To determine the biologically safe concentration of Isorhy, chondrocytes were cultured in different concentrations of Isorhy (0, 5, 10, 20, 25, 50, 100 and 200 μM) for two days. Cell viabilities were analyzed through CCK-8 method. The results showed that chondrocytes were not significantly proliferation-inhibited in prolonged culture with Isorhy lower than 200 μM (Fig. 1). No significant changes in chondrocyte morphology were observed after treatment with 0–200 μM Isorhy matched to the control group.

### Isorhy can activate the expression of genes and proteins related to autophagy and matrix in OA cells

**Fig. 2:**
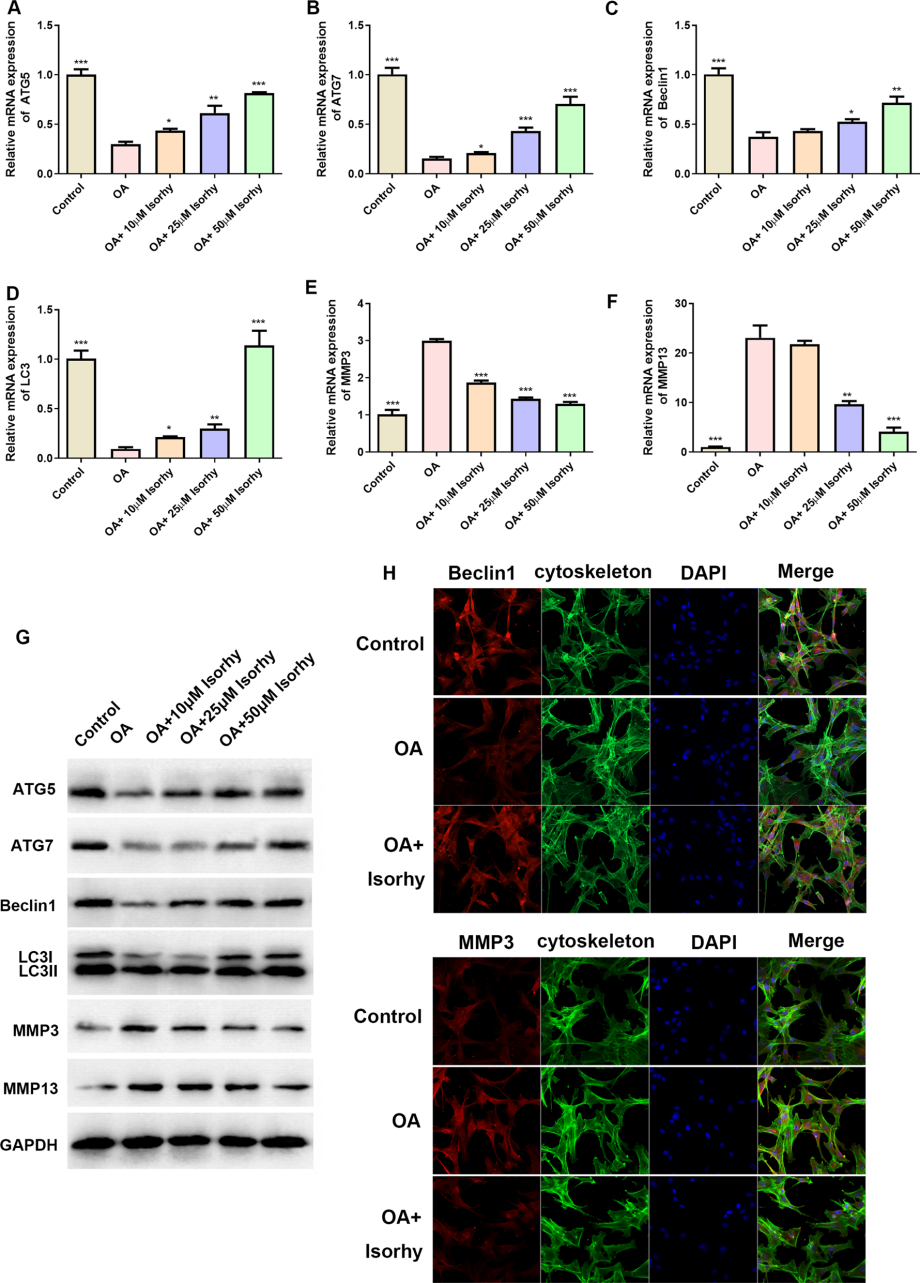
Effects of Isorhy on autophagy and matrix related gene/protein expression **A**–**F** Normal cells, OA cells and OA cells treated with different concentrations of Isorhy were discovered by qRT-PCR for ATG5, ATG7, Beclin-1, LC3, MMP3 and MMP13. **G** Western blotting for ATG5, ATG7, MMP13, Beclin-1, LC3I/LC3II and MMP3 with unequal treatments for 48 h. **H** Immunofluorescent staining for Beclin-1 and MMP3 with unequal conditionsfor 48 h, bar: 100 μm. *P < 0.05, **P < 0.01, ***P < 0.001

C28/I2 chondrocytes was induced by 10 ng/mL IL-1β for 12 h into OA chondrocytes. Due to the significance of autophagy and matrix in OA, the effect of Isorhy on these related proteins were evaluated. ATG5 and ATG7 were involved in autophagosomes elongation steps. Beclin 1 and LC3 play important roles at the autophagosomes formation processes. The results in Fig. 2A–D showed that the mRNA levels of the autophagy-related proteins (such as ATG5, ATG7, Beclin1 and LC3) were significantly decreased in OA chondrocytes compared to ordinary chondrocytes. After that co-incubation of Isorhy, the related mRNA levels were remarkably increased in a dose–effect relationship matched to OA group. In addition, the mRNA expression of matrix-related MMP3 and MMP13 were significantly up-regulated in OA cells compared to normal cells. By Isorhy treatment, the levels of MMP3 and MMP13 were down-regulated compared with the OA group, and the down-regulation showed a certain concentration dependence, and the higher the concentration of Isorhy, the more obvious the down-regulation effect (Fig. 2E and F). Therefore, it can be suggested that Isorhy promoted the gene expression of these proteins in OA chondrocytes but the protein expression should be evaluated to mention that Isorhy generates such an effect. Further, the above findings were confirmed by western blot analysis, which also confirmed that Isorhy restrained the protein expression of MMP3 and MMP13 and advanced the ATG5, ATG7, Beclin-1 and LC3 protein expressions [19] (Fig. 2G). Immunofluorescence staining results in Fig. 2H demonstrated that Isorhy significantly enhanced the level of Beclin-1 while inhibited the level of MMP3 (Fig. 2H).

### Identification of differentially expressed genes and GO enrichment analysis

**Fig. 3:**
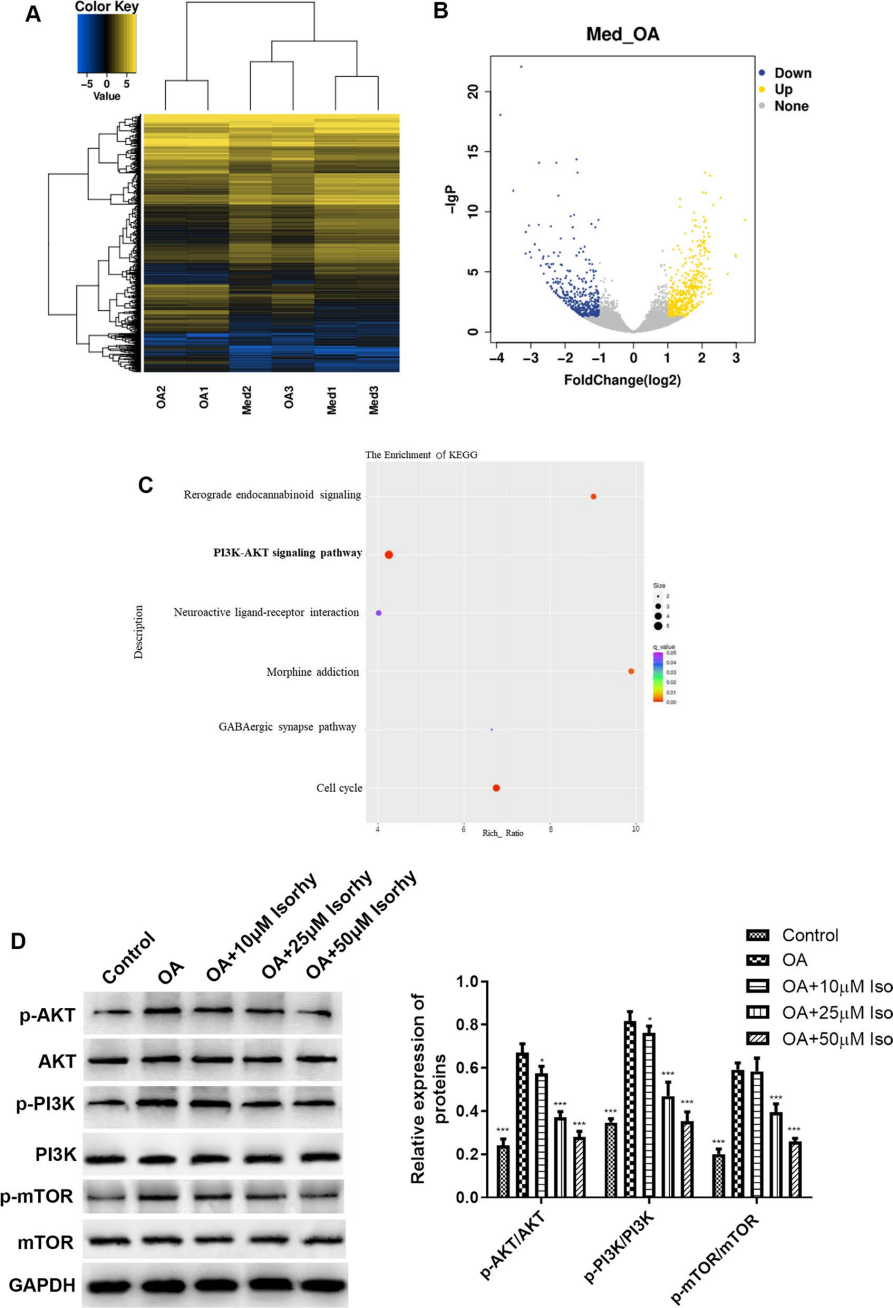
Differential gene expression after treatments under different conditions. **A** Heatmap of gene expression in control group and Isorhy treatment group. **B** Volcano map of gene expression in control group and Isorhy treatment group. **C** KEGG pathway enrichment bubble map. **D** Western blotting and statistical analysis for PI3K, Akt, Mtor, p-PI3K, p-Akt, p-mTOR with different treatments for 48 h, *P < 0.05, ***P < 0.001 versus OA group

To verify the in-depth association of Isorhy in OA chondrocyte, we performed RNA sequencing after different treatment [20]. Normal chondrocytes, OA chondrocytes with and without Isorhy treatment were compared to obtain clustered heat maps and volcano maps of differential gene expression, thus visualizing differential genes (Fig. 3A and B). Enrichment analysis of the KEGG signaling pathway revealed that these variously expressed genes were significantly more abundant in Amoebiasis, Oocyte meiosis and Pyrimidine metabolism, mainly including the PI3K, AKT and mTOR pathway proteins (Fig. 3C). Thus, the expression levels of the PI3K, AKT, mTOR and their respective phosphorylated proteins could be clearly analyzed by Western blotting. The experimental results in Fig. 3D revealed that the different phosphorylation levels of AKT, PI3K and mTOR in OA chondrocytes were fundamentally activated more over that in normal chondrocytes. Isorhy treatment significantly reduced the level of phosphorylated AKT, mTOR and PI3K expressions in a concentration-dependent manner in OA chondrocytes (Fig. 3D), and it confirmed the result of differential gene pathway enrichment.

### Isorhy relieves OA by activating autophagy through inhibiting PI3K/AKT/mTOR signaling in OA chondrocytes

**Fig. 4:**
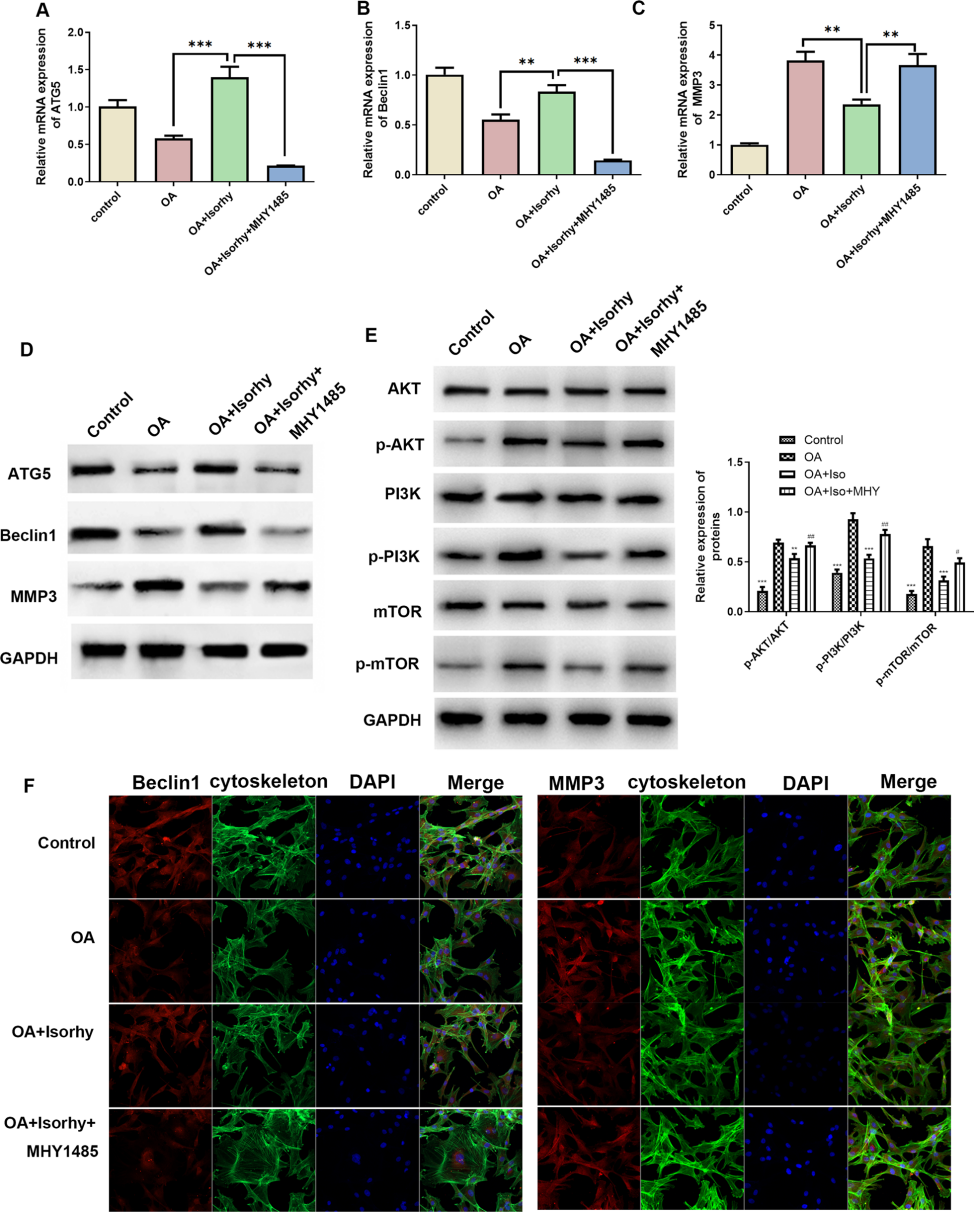
Expression results of related genes/proteins after adding pathway inhibitors [34]. **A**–**C** The mRNA of ATG5, beclin-1 and MMP3 in normal cells, OA cells, OA cells treated with Isorhy and OA cells treated with Isorhy and mTOR inhibitors were detected by qRT-PCR. **D**–**E** Western blot was used to detect the expression levels of PI3K, Akt, mTOR, p-PI3K, p-Akt, p-mTOR, ATG5, beclin-1 and MMP3 in normal cells, OA cells treated with Isorhy and mTOR activator (MHY1485). **F** Immunofluorescence staining results of Beclin-1 and MMP3 after 48 h in four groups of cells, bar: 100 μ m. **P < 0.01, ***P < 0.001 versus OA group, ^#^*P* < 0.05, ^##^*P* < 0.01 versus OA + Isorhy group

Studies have confirmed that the fundamental role of PI3K/AKT/mTOR signaling pathway is advantageous to cell proliferation and inhibit apoptosis, besides its activation plays a key part in strange aspects of cellular life activities. For example, cell proliferation, differentiation, metastasis. Based on the previous researches, we hypothesized that Isorhy could promote autophagy in OA cells via the PI3K/AKT/mTOR pathway. To confirm this hypothesis, we divided the cells into four groups, in order of normal chondrocytes (control), OA chondrocytes [1], OA cells + 50 μM Isorhy (OA + Isorhy) and OA cells + 50 μM Isorhy + MHY1485 (mTOR activator) (OA + Isorhy + MHY1485), respectively. For the above four groups of cells, RT-PCR analysis was performed, and the results showed (Fig. 4A–C) that Isorhy significantly up-regulated the gene expression of ATG5 and Beclin-1 and down-regulated the expression level of MMP3 in OA + Isorhy group compared to that in OA group, and the addition of pathway activator fundamentally down-regulated evidently the gene expression of the ATG5 and the Beclin-1, in addition up-regulated evidently the expression level of MMP3. Western blot (Fig. 4D) and immunofluorescence staining results (Fig. 4F) were consistent with the above results. We also analyzed the expression levels of PI3K, AKT, mTOR and their respective phosphorylated proteins in cells with or without the addition of MHY1485, and confirmed that Isorhy can suppress the expression of phosphorylated PI3K, AKT, and mTOR, while MHY1485 rescued this effect, that upregulated the phosphorylation levels (Fig. 4E). All of these findings suggested that Isorhy relieves OA by activating autophagy through inhibiting PI3K/AKT/mTOR signaling in OA chondrocytes.

### Isorhy alleviated OA in rat OA models

**Fig. 5:**
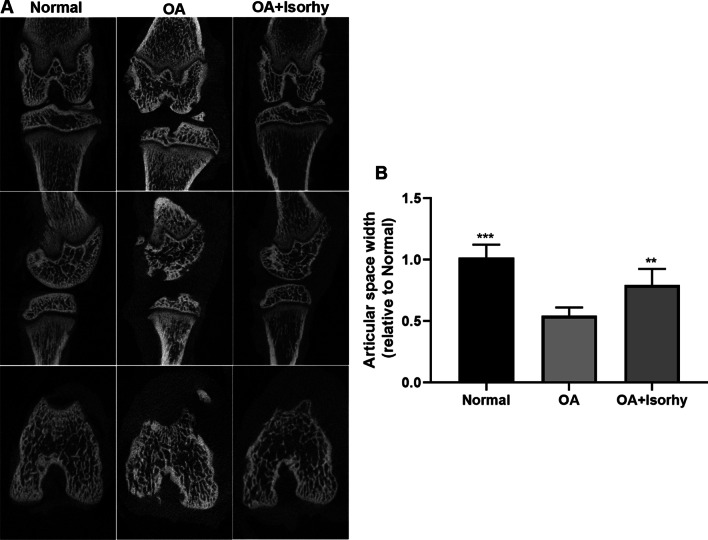
Isorhy reduced the reduction of joint space in vivo. **A** Typical micro-CT images of normal cells, OA cells and OA cells after Isorhy treatment, **B** The relative articular space width of three groups, which is determined from the micro-CT images. **P < 0.01, ***P < 0.001

In addition, we also confirmed the above idea through animal experiments that Isorhy cures OA in rat OA models. From the experimental results, it is distinct that the micro-CT images of the different groups rat knee joint after treatments in Fig. 5A indicated that the geomorphology of all rat knee joint. The joint space of OA group turns narrower after the rat knee joint surgery compared to normal group. The current result shows that the accurate establishment of the rat OA model by the DMM surgery. Fundamentally, the joint space width of OA + Isorhy group is well above that of OA group, suggesting that Isorhy is beneficial to inhibit the development of OA in vivo.

**Fig. 6:**
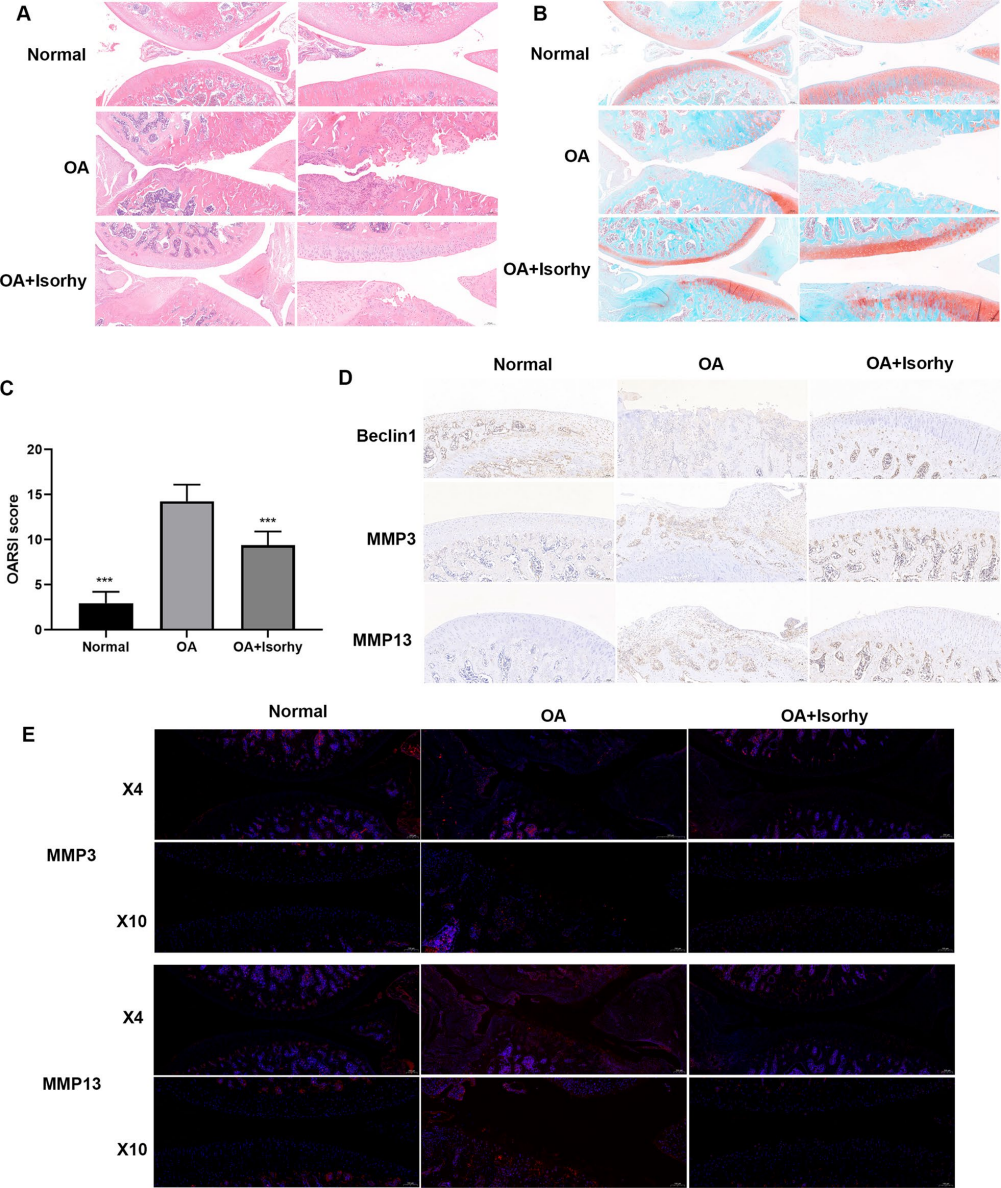
Histological analysis of knee cartilage after Isorhy treatment (H&E, safranin-O/green, and IF). **A** Typical images of hematoxylin–eosin staining, **B** Images of Safranin O-fast green staining for different groups. **C** OARSI score of articular cartilage in different groups of rats. **D** The expression level of Beclin1, MMP3 and MMP13 determined by immunohistochemical. **E** The expression level of MMP3, MMP13 decided by immunofluorescence. ***P < 0.001

The cartilage tissue of the mice was treated for histological evaluation. The typical images of HE staining (Fig. 6A) and Safranin O-Fast green staining (Fig. 6B) proved serious cartilage damage in the OA group compared to the normal group, and intra-articular treatment of Isorhy evidently mitigate surgical resection-induced cartilage damage compared with the OA group. What’s more, tt is noted OARSI score (Fig. 6C) of OA group was significantly higher than that of narmal group, and Isorhy treatment decreased greatly the score compared with OA group.

The results of immunohistochemical (Fig. 6D) and immunofluorescence (Fig. 6E) also revealed that Isorhy increased the expression of Beclin1 and decreased the levels of MMP3 and MMP13. All the above indicated Isorhy can effectively improve OA, promote autophagy, and reduce cartilage matrix degradation in rat OA models.

## Discussion

Osteoarthritis is a familiar ailment of the locomotor system characterized thought chondrocyte wreck, and its incidence rate is categorically correlated with age [6, 7]. Until now, the exact etiology and pathogenesis are still unclear and need to be further investigated [18, 21, 22]. Autophagy maintains chondrocyte homeostasis in normal adult articular cartilage, and the chondrocytes in the superficial layer of articular cartilage highly express Beclin-1, ATG5 and LC3 II. In addition, autophagy of chondrocytes will also increase in the initial stage of metabolism and starvation stress. Therefore, in the initial stage of articular cartilage degeneration, the expression of LC3 II and Beclin-1 in OA chondrocytes increased [23]. In the rat model of temporomandibular arthritis, the early degenerative articular chondrocytes were observed by transmission electron microscope, and the autophagy bodies increased; With the decrease of mTOR activity, the transcription and expression of LC3 II and Beclin-1 increased [24]. The transient increase of autophagy is a compensatory response of cells to stress, which is conducive to cell survival. If autophagy increases continuously, cells will be damaged and apoptosis [23, 25]. Chondrocytes with defective mitochondrial autophagy exhibit pathological features such as apoptosis, loss of extracellular matrix and other degenerative changes of OA. PI3K/AKT/mTOR, as one of the classical tumors signaling pathways, has been shown to regulate both apoptosis and autophagy to exert anti-tumor effects. Isorhy is an indole alkaloid found in the Chinese herbal medicine, and it is the main active ingredient of Crocus sativus. It has been shown that it can induce autophagic death in human glioma cells.

In this paper, we analyzed the classical pathway effect of Isorhy on apoptosis, and thus confirmed that Isorhy plays a key role in the treatment of OA cells. Thus, we first confirmed the biological safety of is Isorhy. Apoptosis and autophagy are two important manifestations of programmed cell death, which have an important role in tumor development and treatment process [26]. LC3 protein is one of the markers of autophagy. When autophagy occurs, LC3I is converted to LC3 II, and the level of autophagy can be estimated by evaluating the ratio of LC3 II/LC3 I [22]. An increase in LC3 II expression indicates that the expression level of autophagy is upregulated, while the opposite is downregulated. ATG5 is also an important marker of autophagy along with ATG7, which encodes a ubiquitin E1-like ligase that activates ATG12 prior to its binding to ATG5 and promotes the expansion of the pro-phagosome [27, 28]. MMP-1 and MMP-13 are rarely expressed in normal chondrocytes, while their expression is significantly elevated in OA cells. Thus, Isorhy treatment activated OA cell autophagy with upregulated expression levels of ATG5, ATG7, LC3 and Beclin-1. Besides, downregulated expression levels of MMP-1, MMP-13.

As PI3K combines to growth factor receptors (EGFR), it can alter the composition of downstream AKT and activate it, affecting a series of downstream substrates for example apoptosis-associated protein Bad and Caspase-9 activity, thus regulating cell proliferation, apoptosis and autophagy. The mTOR is a downstream regulator of PI3K/AKT, which can regulate several life activities and has a crucial part in regulating apoptosis and autophagy [29–31]. The mTOR plays a negative feedback role in regulating autophagy and a dual role in apoptosis. As one of the classical tumors signaling pathways, PI3K/AKT/mTOR has been shown to regulate both asis and autophagy to exert anti-tumor effects. In the current research, we seriously compared the results of differentially expressed genes with protein expression in the database and concluded that Isorhy treatment of OA cells significantly downregulated the proteins expression of p-AKT, p-PI3K and p-mTOR [32, 33]. Next, during this study, the mTOR pathway agonist MHY1485 was added along with Isorhy interference, and both results showed a significant decrease in OA cell autophagy after the combination of the two. Finally, the present study reconfirmed that Isorhy activates OA cell autophagy by blocking PI3K/AKT/mTOR signaling pathway effectively in clinical mice, thereby reducing the inflammatory response.

In summary, we found that Isorhy promotes the autophagic process thought restraining the PI3K/AKT/mTOR signaling pathway. Our study investigated that the novel protective mechanism of Isorhy in osteoarthritis and indicated that Isorhy may become a new potential therapeutic target for osteoarthritis.

## Conclusion

This study shows the PI3K/AKT/mTOR signaling pathway is really a critical pathway for cellular autophagy and that Isorhy promotes autophagy in OA cells thought inhibiting the PI3K/AKT/mTOR signaling pathway precisely, thereby effectively alleviating the progression of osteoarthritis.

## Data Availability

The data sets used and/or analyzed during the current study are available from the corresponding author on reasonable request.

## Abbreviations

Isorhy: Isorhynchophylline
OA: Osteoarthritis
FBS: Fetal bovine serum
CCK-8: Cell counting kit 8
OD: Optical density
qRT-PCR: Quantitative real time PCR
PBS: Phosphate buffer saline
